# Diffuse intra-abdominal splenosis presenting as carcinomatosis exhibiting positron emitted tomography hypermetabolic activity^☆^

**DOI:** 10.1016/j.gynor.2013.03.004

**Published:** 2013-04-02

**Authors:** Brian Kellert, Michelle Caster, Ryan Des Jean, Luis Vaccarello

**Affiliations:** a The Ohio State University/Mount Carmel Health OB/GYN Residency Program, 395 W State St, 5th Floor, Columbus, OH 43210, United States; b Mount Carmel Health System, Mount Carmel East Hospital, Department of Pathology, 5959 E Broad Street, Columbus, OH 43213, United States; c The Zangmeister Cancer Center, 3100 Plaza Properties Blvd, Columbus, OH 43219, United States

**Keywords:** Splenosis, Carcinomatosis, PET

## Abstract

• Splenosis can mimic carcinomatosis upon many imaging modalities.
• History of splenectomy must be considered when evaluating carcinomatosis.
• Scintigraphy is the preferred for confirming the presence of splenosis.

## Case report

CL is a 54 year old perimenopausal, nulliparous, morbidly obese Caucasian woman who was referred to gynecologic oncology after discovery of peritoneal implants on CT scan of the abdomen and pelvis, obtained for persistent epigastric pain. She denied history of weight loss, bloating, constipation, pelvic pain, or dysuria. Her history included laparoscopic splenectomy thirteen years prior for idiopathic thrombocytopenic purpura (ITP), and she had no family history of cancer.

**Fig. 1:**
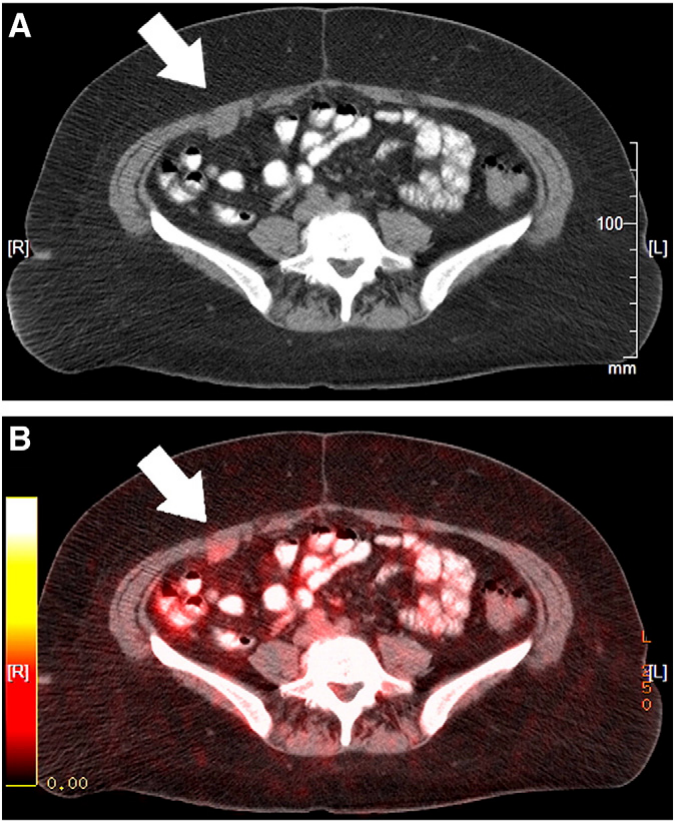
A: CT scan with IV contrast, coronal section at L5 showing anterior peritoneal wall implants on the patient’s right. B: PET scan showing metabolic hyperactivity in these same implants.

Abdominal and rectopelvic examinations were normal. Cancer antigen 125 and carcinoembryonic antigen levels were within normal limits. CT review identified multiple small enhancing anterior peritoneal nodular implants without evidence of pelvic mass (Fig. 1A). Given pronounced imaging findings, resolution of prior symptoms, and history of splenectomy, a positron emission tomography (PET) scan was obtained and showed hypermetabolic activity within some implants (Fig. 1B).

**Fig. 2:**
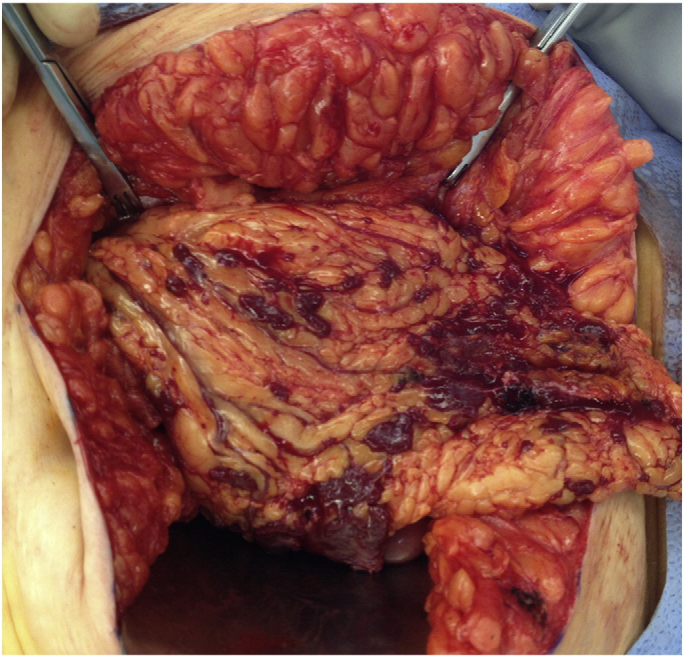
Intraoperative view of anterior surface of greater omentum with splenosis implants.

As the case was highly suspicious for a malignant process, the patient was consented for exploratory laparotomy and debulking. Intraoperatively, the greater omentum had multiple small maroon nodules under 3 cm in size (Fig. 2). Extensive implantation was observed along the anterolateral abdominal walls, small and large bowel, bladder, uterus, ovaries, and tubes. A partial omentectomy was performed and sent for frozen and permanent section. Pathologist review of frozen sections found lymphoid tissue favoring spleen which confirmed our clinical suspicions. Complete survey found otherwise normal abdominopelvic organs, and the abdomen was closed. The final pathology report described unremarkable spleen, confirmed by flow cytometry revealing normal lymphoid populations. When the patient returned to the office for post-operative examination, she had a peripheral blood smear that showed no Howell–Jolly bodies.

We reviewed her splenectomy operative report which described laparosocpic excision with morcellation and removal through an endoscopic bag without spillage. No comment was made of splenic nodules. The patient's platelet count four months prior to splenectomy was 21 K/μL, rising to 383 K/μL the day of splenectomy with medical therapy, then rising to 583 K/μL one month following surgery. Her platelet count was 432 K/μL at the time of consultation thirteen years later.

## Discussion

Splenosis is defined as ectopic splenic tissue commonly arising from splenic trauma-induced autotransplantation, or rarely as congenital accessory spleens or polysplenia (Fremont and Todd, 2007; Lake et al., 2012). Ninety three percent of splenosis cases follow abdominal trauma, and arises after 65% of splenic ruptures (Fremont and Todd, 2007; Ksiadzyna and Peña, 2011; Malik et al., 2012). Small (< 3 cm) visceroperitoneal implants are typically found, and secondary splenosis is associated with greater implant quantity (maximum of 400 reported) compared to congenital origins (up to 10) (Fremont and Todd, 2007; Ksiadzyna and Peña, 2011). Implants may arise months to years following initial insult, and may be functional such as in recurrence of ITP (Ksiadzyna and Peña, 2011). These implants may mimic carcinomatosis upon front line imaging modalities (Neri et al., 1986; White et al., 1989; Short et al., 2011; Stovall and Ling, 1988; Mikhael et al., 2009).

The recurrence risk of ITP secondary to splenosis is unclear. Meta-analysis of 23 reports of ITP relapse following laparoscopic splenenectomy (74% performed for ITP) was 43.6 per 1000 patient years, 1–5 years post-operatively (Mikhael et al., 2009). Khatkouda et al. compare their post-splenectomy ITP recurrence risk of 6% to the 4–24.3% range of several studies, and suggest that capsular rupture may increase recurrence to 12.5% (Katkhouda et al., 1998). The lack of Howell–Jolly bodies 13 years post-splenectomy suggests functional splenotic nodules in our patient, thus a risk of ITP recurrence exists.

The majority of splenosis cases are found incidentally, and it has rarely been reported as a primary pathologic process. As concisely reviewed by Fremont et al.; “Pain secondary to infarction, intestinal obstruction due to the adhesive bands of the splenic implants, gastrointestinal hemorrhage, hydronephrosis secondary to a growing mass exerting pressure on the ureter, as well as an enlarging abdominal mass with associated infection have all been reported (Fremont and Todd, 2007).”

Imaging modalities such as ultrasound, CT, and MRI do not provide confirmatory diagnosis. Splenosis may resemble normal splenic tissue upon these imaging techniques, and none can exclude carcinomatosis (Lake et al., 2012; Ksiadzyna and Peña, 2011). Many radiologic and nuclear imaging techniques have been explored for non-invasive confirmation of suspected splenosis, with 99 m Technetium heat-damaged erythrocyte scintigraphy regarded as ideal due to preferential uptake in splenic tissue with normal liver uptake (Ksiadzyna and Peña, 2011; Malik et al., 2012).

PET scans assist oncologists in treatment planning and malignancy response quantification. Injection of labeled biologically active glucose results in concentration of tracer in many tissues exhibiting increased metabolic activity, a fundamental process in malignant tumors (Aluddin, 2012). Quantification of glucose metabolism is dependent upon many variables, including basal glucose levels, stress, volume of distribution, medications, in addition to standardized and facility-specific protocols (Vriens et al., 2010).

A literature review found no reports of splenosis exhibiting PET hypermetabolic activity. In fact, a recent case report of peritoneal splenosis mimicking carcinomatosis used negative PET findings in support of primary laparoscopy instead of laparotomy in a patient with history of cervical cancer (Ake et al., 2012). While PET signal may be influenced by habitus and glucoregulation, this morbidly obese patient was not diabetic, fasted properly prior to the study, had a normal glucose level (93 mg/dL), and underwent a standardized protocol with fused PET–CT images and appropriate uptake phase of 60 min.

Direct visualization and tissue biopsy are the ideal means of characterizing a suspected malignant intra-abdominal process. This patient had normal tumor marker levels and resolution of symptoms, but ominous CT and PET findings. Given this clinical picture, we proceeded with planned laparotomy for debulking surgery. Her surgery and recovery were uncomplicated and the findings provided her with great relief. In retrospect, scintigraphy may have added information to suggest a preoperative diagnosis of splenosis. In this case, visual and tissue confirmation could have been accomplished by laparoscopy. In this patient's clinical scenario we feel that visual and pathologic confirmation is mandatory. The vascular nature of splenic implants may increase the risk for hemorrhage with CT or ultrasound-guided biopsy, and this method would not provide for visual survey of the abdominopelvic cavity.

## Conclusions

Splenosis is a rare phenomenon that may be mistaken for carcinomatosis upon standard imaging techniques. PET is routinely used to better characterize an abdominopelvic process suspicious for malignancy. In workup of novel carcinomatosis in patients with history of abdominal trauma or splenectomy, splenosis should be part of the differential diagnosis, and consideration of nuclear imaging modalities including PET scan, scintigraphy, and diagnostic laparoscopy should all be considered prior to proceeding with laparotomy.

## Conflict of interest statement

The authors of this manuscript have no conflicts of interests to disclose.

## Consent

Written informed consent was obtained from the patient for publication of this case report and accompanying images. A copy of the written consent is available for review by the Editor-in-Chief of this journal on request.
